# Analysis of Gene Expression Profiling in Meningioma: Deregulated Signaling Pathways Associated with Meningioma and EGFL6 Overexpression in Benign Meningioma Tissue and Serum

**DOI:** 10.1371/journal.pone.0052707

**Published:** 2012-12-28

**Authors:** Xuanchun Wang, Ye Gong, Daijun Wang, Qing Xie, Mingzhe Zheng, Yu Zhou, Qin Li, Zhen Yang, Hailiang Tang, Yiming Li, Renming Hu, Xiancheng Chen, Ying Mao

**Affiliations:** 1 Department of Endocrinology, Huashan Hospital, Shanghai Medical College, Fudan University, Shanghai, China; 2 Department of Neurosurgery, Huashan Hospital, Shanghai Medical College, Fudan University, Shanghai, China; Beijing Tiantan Hospital, Capital Medical University, China

## Abstract

Molecular mechanisms underlying the pathogenesis of meningioma are not fully elucidated. In this study, we established differential gene expression profiles between meningiomas and brain arachnoidal tissue by using Affymetrix GeneChip Human U133 Plus 2.0 Array. KEGG pathway analysis demonstrated that PI3K/Akt and TGFβ signaling pathways were up-regulated in fibroblastic meningioma, and focal adhesion and ECM-receptor interaction pathways were activated in anaplastic meningioma. *EGFL6* was one of the most up-regulated genes in fibroblastic meningioma by microarray analysis. Quantitative real-time PCR demonstrated that benign meningiomas had significantly higher levels of *EGFL6* mRNA than brain arachnoidal tissue and atypical and anaplastic meningiomas (*P*<0.001). *EGFL6* gene was also highly expressed in ovarian cancer, but expressed lowly in other investigated tumors. ELISA analysis showed that patients with benign meningiomas and ovarian cancers had the highest serum levels of EGFL6 (mean concentration: 672 pg/ml for benign meningiomas, and 616 pg/ml for ovarian cancers). Healthy people and patients with other tumors, however, had low levels of serum EGFL6. In conclusion, we proposed that activation of PI3K/Akt and integrin-mediated signaling pathways was involved in the pathogenesis of benign and anaplastic meningiomas, respectively. We also presented evidence that EGFL6 was overexpressed in benign meningioma tissues and serum.

## Introduction

Meningiomas are mesenchymal tumors that arise from the arachnoidal cells of the leptomeninges covering the brain and spinal cord, and account for approximately 30% of all primary intracranial neoplasms [1]. According to the WHO grading system, these tumors are classified as benign WHO grade I (approximately 80% of meningiomas), atypical WHO grade II (5% to 20%), and anaplastic WHO grade III (1% to 2%). Based on histopathological characteristics of tumor cell, grade I meningiomas are classified into different subtypes such as meningiothelial, fibroblastic, transitional, psammomatous, angiomatous, and secretory meningiomas [2], [3], [4].

As for mechanisms underlying the pathogenesis of meningioma, cytogenetic studies have revealed several chromosomal abnormalities in meningioma including losses of 1p, 6q, 9p, 10p, 10q, 14q and 18q, and gains of 1q, 9q, 12q, 15q, 17q and 20q [1]. The inactivation of the *NF2* gene at 22q12 has been identified as one of the most common events associated with meningioma tumorigenesis [5], [6]. It has been reported that the majority of anaplastic meningiomas either show homozygous deletions of *CDKN2A*, *p14^ARF^*, and *CDKN2B*, mutations in *CDKN2A* and *p14^ARF^*, or lack of expression of one or more of these genes [7]. Recently, *MEG3* gene was observed loss of its RNA expression as well as loss of its gene copy number in higher-grade meningioma and an overall increase in CpG methylation in tumors associated with tumor grade [8].

Clinicopathological examination of meningioma has benefited from the discovery of several molecular markers such as vimentin and epithelial membrane antigen [2]. Presence of other molecular markers in meningioma has been reported recently. Aruga et al [9] revealed that Zic family genes including *ZIC1*, *ZIC2*, and *ZIC5* were highly expressed in meningioma, suggesting these Zic proteins as novel molecular markers for meningioma. In another study, Saydam et al [10] found that the minichromosome maintenance (MCM) family proteins were highly and significantly up-regulated in meningioma samples compared to arachnoidal tissues, implying that MCMs can serve as diagnostic biomarkers for meningioma.

We have established differential gene expression profiles between meningioma and brain arachnoidal tissue by using cDNA microarray. In the current study, we attempted to search for novel molecular mechanisms implicated in the tumorigenesis of meningioma by using KEGG pathway analysis on the differentially expressed genes between arachnoidal tissue and meningioma. We also presented evidence that EGFL6, a secreted protein, belonging to the epidermal growth factor (EGF) repeat superfamily, was overexpressed in benign meningioma tissues and serum.

## Materials and Methods

### Ethics statement

Informed consent written by all participants was obtained, and studies on brain arachnoidal tissue, various tumor samples, and human sera were approved by the Human Subjects Institutional Review Boards at Huashan Hospital, Shanghai, China.

### Patients and collection of serum samples

Patients with various tumors hospitalized at Huashan Hospital, Fudan University, Shanghai, China, were enrolled in this study. Two hundred age- and gender-matched healthy people from the center of physical examination at Huashan Hospital were also included in the study. These patients were clinically diagnosed and afterwards confirmed by pathological examinations of tumor samples. Before these patients were performed surgeries or received any therapies, three-milliliter samples of blood were collected, and then serum samples were separated and preserved at −80°C until used. Control serum samples were collected from those healthy people.

### Tissue samples

Tumor samples were obtained from patients undergoing surgery at Huashan Hospital. Tumor tissues were snap-frozen in liquid nitrogen at the time of surgery and stored at −80°C until used. H&E stained frozen sections were prepared from tumor samples to obtain information about histological subtype and histopathological grade. Brain arachnoidal tissue was generously supplied by Shanghai Body Donation Center, Shanghai, China.

### Microarray analysis

Total RNA was extracted from 3 brain arachnoidal tissues, 3 fibroblastic meningiomas, and 3 anaplastic meningiomas by using Trizol Reagent (Invitrogen), and then purified with RNeasy Micro kits (Qiagen). RNA quality and yield were assessed on an Agilent Bioanalyzer 2100 (Agilent Technologies). For the first-round synthesis of double-stranded cDNA, total RNA was reverse-transcribed using the One-Cycle cDNA Synthesis Kit (Affymetrix). After purification of cDNA with a GeneChip Sample Cleanup Module column (Affymetrix), second-round double-stranded cDNA was amplified using the GeneChip IVT Labeling Kit (Affymetrix) to produce labeled cRNA. Labeled product was fragmented, and then hybridized to the Affymetrix GeneChip Human U133 Plus 2.0 Array. Finally, labeled cRNA was stained on a GeneChip Fluidics Station 450 (Affymetrix) and scanned on a GeneChip Scanner 3000 7G (Affymetrix). Scanned raw data images were processed with GeneChip Operating Software (GCOS) 1.4. A quality control report was subsequently made using Bioconductor, and the data were modeled using the RMA (Robust Multichip Average) approach [11]. Microarray data are MIAME compliant and available in Gene Expression Omnibus (GEO, http://www.ncbi.nlm.nih.gov/geo/) through the accession number GSE32197. Differentially expressed genes were identified by setting the significance level to a false discovery rate of <0.1. Comparing the mean expression levels of the groups, the threshold level was set at >2 and <0.5-fold differences.

### KEGG pathway analysis

In this study, we used Kyoto Encyclopedia of Genes and Genomes (KEGG) pathways as the primary tool to identify potential gene pathways that may be involved in the pathogenesis of meningioma. KEGG pathway analysis was performed by using the Database for Annotation, Visualization and Integration Discovery (DAVID) Knowledgebase (http://david.abcc.ncifcrf.gov/) [12]. The differentially expressed (>2 or <0.5-fold) genes between brain arachnoidal tissue and meningioma were input and the significance level for the pathway analysis was set as *P* value <0.01.

### Quantitative real-time PCR

Validation of microarray results was carried out using the QuantiTect SYBR green PCR kit (Qiagen), as recommended, in a MicroAmp Optical 96-well reaction plate by using a 7500 Sequence Detector (Applied Biosystems). The relative quantification of the target gene at transcriptional level in each sample was expressed in 2^−ΔCT^. ΔCT corresponds to the difference in CT between the target gene and the internal control gene (human *GAPDH*). All qRT-PCR experiments were performed in duplicate.

### Western blot analysis

Total proteins were extracted from meningioma samples and brain arachnoidal tissue by using RIPA buffer (Pierce). Antibodies for western blotting included rabbit anti-Akt polyclonal antibody (Santa Cruz Biotechnology) (1∶1000), rabbit polyclonal antibody against p-Akt (Thr 308) (Santa Cruz Biotechnology) (1∶1000), rabbit anti-FAK polyclonal antibody (Cell Signaling Technology) (1∶1000), rabbit polyclonal antibody against phospho-FAK (Tyr 397) (Cell Signaling Technology) (1∶1000), and rabbit monoclonal antibody against GAPDH (Cell Signaling Technology) (1∶5000). Western blotting was performed as described previously [10].

### Measurement of serum EGFL6

The serum EGFL6 was determined in duplicate by ELISA kits (Uscn Life Science Inc.) as recommended by the manufacturer. The ELISA system had an intra-assay coefficient of variation of 3–8% and an inter-assay coefficient of variation of 4–10%, respectively.

### Statistical analysis

Data were presented as means ± SEM. Statistical analysis was performed by using SPSS software (version 13.0; SPSS Inc). The independent samples *t* test was used to determine the differences in serum EGFL6 concentrations, *EGFL6* gene expression, and expression of genes in the KEGG pathways among different groups. *P*<0.05 was considered significant. Pearson correlation coefficients and related *P* values were calculated to evaluate the association between serum EGFL6 levels and meningioma sizes. Receiver operating characteristics (ROC) curve was constructed to assess sensitivity, specificity, areas under the curve (AUC), and cutoff point of serum EGFL6 concentration.

## Results

### KEGG pathways in fibroblastic meningioma

We used KEGG pathway analysis for discovering differentially expressed gene pathways between meningioma and brain arachnoidal tissue, as the primary tool to identify signaling pathways associated with tumorigenesis of meningioma. Firstly, KEGG pathways in fibroblastic meningioma were determined. Totally, 13 pathways with *P* value less than 0.01 were obtained, of which pathway in cancer (hsa05200) was the most significant pathway in fibroblastic meningioma (Table S1). According to the differentially expressed genes, we figured out three sub-pathways that may be involved in the pathogenesis of fibroblastic meningioma, including PI3K/Akt, cell cycle, and TGFβ signaling pathways (Figure S1).

**Figure 1:**
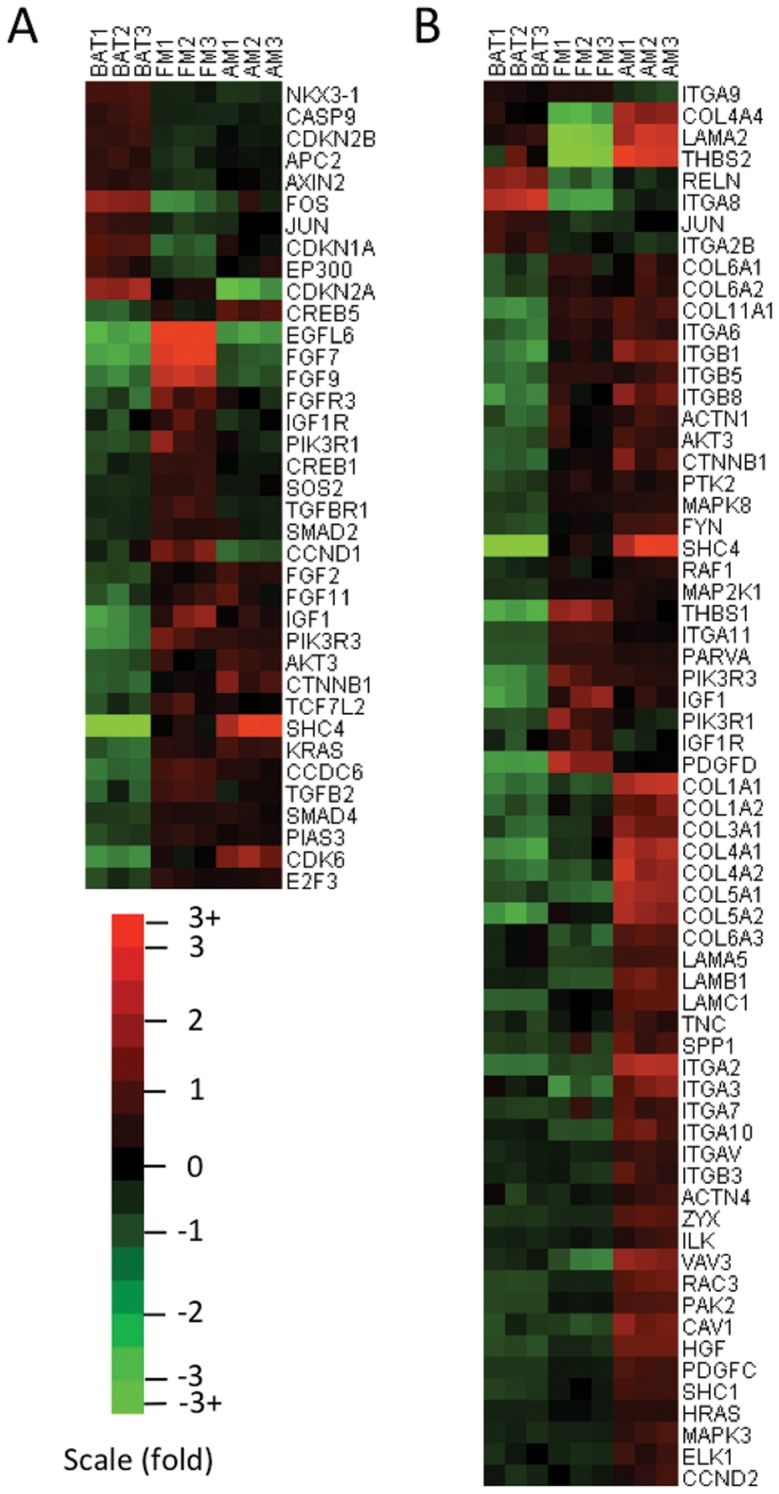
Relative expression of genes associated with KEGG pathways by microarray analysis. (A) Expression of genes associated with KEGG pathways of fibroblastic meningioma. (B) Expression of genes involved in KEGG pathways of anaplastic meningioma. Key words: BAT, brain arachnoidal tissue; FM, fibroblastic meningioma; AM, anaplastic meningioma.

**Figure 2:**
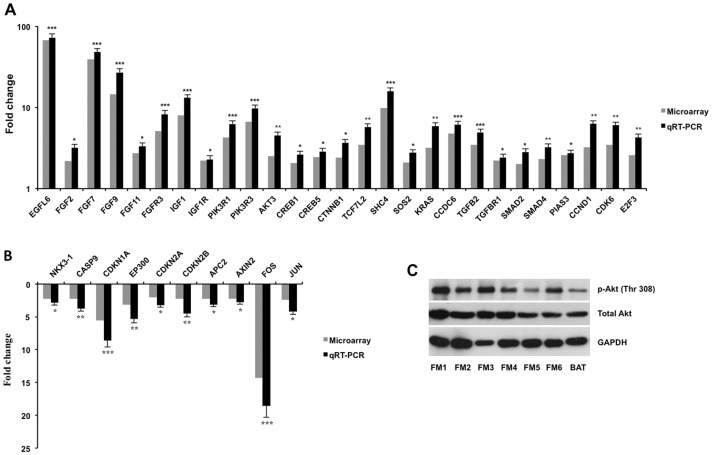
Expression of genes/proteins associated with the KEGG pathways in fibroblastic meningiomas and arachnoidal tissue. qRT-PCR experiments were performed to confirm the expression of genes in 10 fibroblastic meningiomas (including the 3 tested samples in microarray) and 3 arachnoidal tissues. When expression of the genes in arachnoidal tissue was set to 1, up-regulated (A) and down-regulated (B) folds of the genes in fibroblastic meningiomas are shown in each graph by using microarray and qRT-PCR. *P<0.05, **P<0.01, ***P<0.001. Western blotting determined total Akt and phosphorylated Akt (p-Akt Thr 308) in fibroblastic meningiomas and arachnoidal tissue (C). GAPDH expression was used as controls. Key words: FM, fibroblastic meningioma; BAT, brain arachnoidal tissue.

Expression of those genes associated with KEGG pathways of fibroblastic meningioma evaluated by microarray experiments was shown in a heat map (Figure 1A), and qRT-PCR experiments were performed to confirm the microarray results. We first investigated differential expression of growth factors and their receptors between tumor and arachnoidal tissue. Significantly, *EGFL6* expression in fibroblastic meningiomas increased 68- and 73-fold by microarray and qRT-PCR respectively when compared with arachnoidal tissue (Figure 2A). Several fibroblast growth factor (FGF) family genes were also found up-regulated in tumor tissues. Expression of *FGF2*, *FGF7*, *FGF9*, and *FGF11* was significantly increased 3.19-, 48.32-, 27.15-, and 3.34-fold respectively in fibroblastic meningiomas compared with arachnoidal tissue by qRT-PCR (Figure 2A). Of FGF receptors, only *FGFR3* was found with significantly enhanced expression in tumor tissues (8.21-fold, vs arachnoidal tissue) (Figure 2A). Other FGF receptors such as *FGFR1*, *FGFR2*, and *FGFR4* had no significant differences in expression between tumor and arachnoidal tissue (data not shown). Additionally, qRT-PCR analysis indicated 13.17- and 2,28-fold up-regulation of *IGF1* and its receptor (*IGF1R*) respectively in tumor tissues (Figure 2A). It is well known that up-regulation of the growth factor-receptor interaction exerted impacts on tumor growth through the downstream PI3K/Akt pathway. We found that expression of *PIK3R1*, *PIK3R3*, and *AKT3* significantly enhanced 6.27-, 9.78-, and 4.53-fold respectively in tumor tissues in comparison with arachnoidal tissue by qRT-PCR (Figure 2A). Western blot analysis showed that both total Akt and phosphorylated Akt obviously increased in fibroblastic meningiomas compared with brain arachnoidal tissue (Figure 2C). *NKX3-1*, a negative regulator of Akt, was observed down-regulation of 2.18-fold in tumor tissues (Figure 2B). Regarding downstream gene cascades of Akt, apoptosis-induced genes including *CASP9* (3.78-fold), *CDKN1A* (8.64-fold), *AXIN2* (2.78-fold), *APC2* (3.12-fold), and *EP300* (5.35-fold) were detected with significant down-regulation in fibroblastic meningiomas (Figure 2B). On the contrary, proliferation-promoted genes such as *CTNNB1*, *CREB1*, *CREB5*, and *TCF7L2* were up-regulated 3.65-, 2.63-, 2.87-, and 5.75-fold respectively in tumor tissues (Figure 2A). It is well established that Ras/ERK pathway is one of the most important downstream components of growth factor-receptor interaction. In our study, qRT-PCR analysis showed *SHC4* (15.85-fold), *SOS2* (2.78-fold), *CCDC6* (6.18-fold), and *KRAS* (5.92-fold) were up-regulated significantly in tumor tissues (Figure 2A). Contradictorily, c-Jun (4.21-fold) and c-Fos (18.56-fold), two important downstream targets of Ras/ERK pathway, were observed with significantly decreased expression at mRNA levels in tumor tissues (Figure 2B). TGFβ signaling pathway was also found activated in fibroblastic meningioma, since multiple key genes in the pathway were up-regulated in tumor tissues by qRT-PCR, including *TGFB2* (4.95-fold), *TGFBR1* (2.43-fold), *SMAD2* (2.82-fold), and *SMAD4* (3.24-fold) (Figure 2A). As for genes associated with cell cycle pathway, the results of qRT-PCR showed up-regulation of *CCND1* (6.28-fold), *CDK6* (6.03-fold), and *E2F3* (4.26-fold) in fibroblastic meningiomas, and down-regulation of *CDKN1A* (8.64-fold), *CDKN2A* (3.21-fold), and *CDKN2B* (4.53-fold), suggesting accelerated G1/S progression and promoted proliferation of tumor cells (Figure 2A, 2B).

### KEGG pathways in anaplastic meningioma

Totally, twelve significant (*P*<0.01) pathways in anaplastic meningioma were generated (Table S2). Of these pathways, focal adhesion (hsa04510) and ECM-receptor interaction pathway (hsa04512) had the lowest *P* values as 2.35E-07 and 7.10E-07 respectively, indicating the highest significance levels. Importantly, most of these genes associated with the two pathways were up-regulated in anaplastic meningioma compared with normal arachnoidal tissue, suggesting activation of focal adhesion and ECM-receptor interaction pathways in anaplastic meningioma (Figure S2). A heat map was also established to indicate expression of genes involved in the two pathways (Figure 1B).

**Figure 3:**
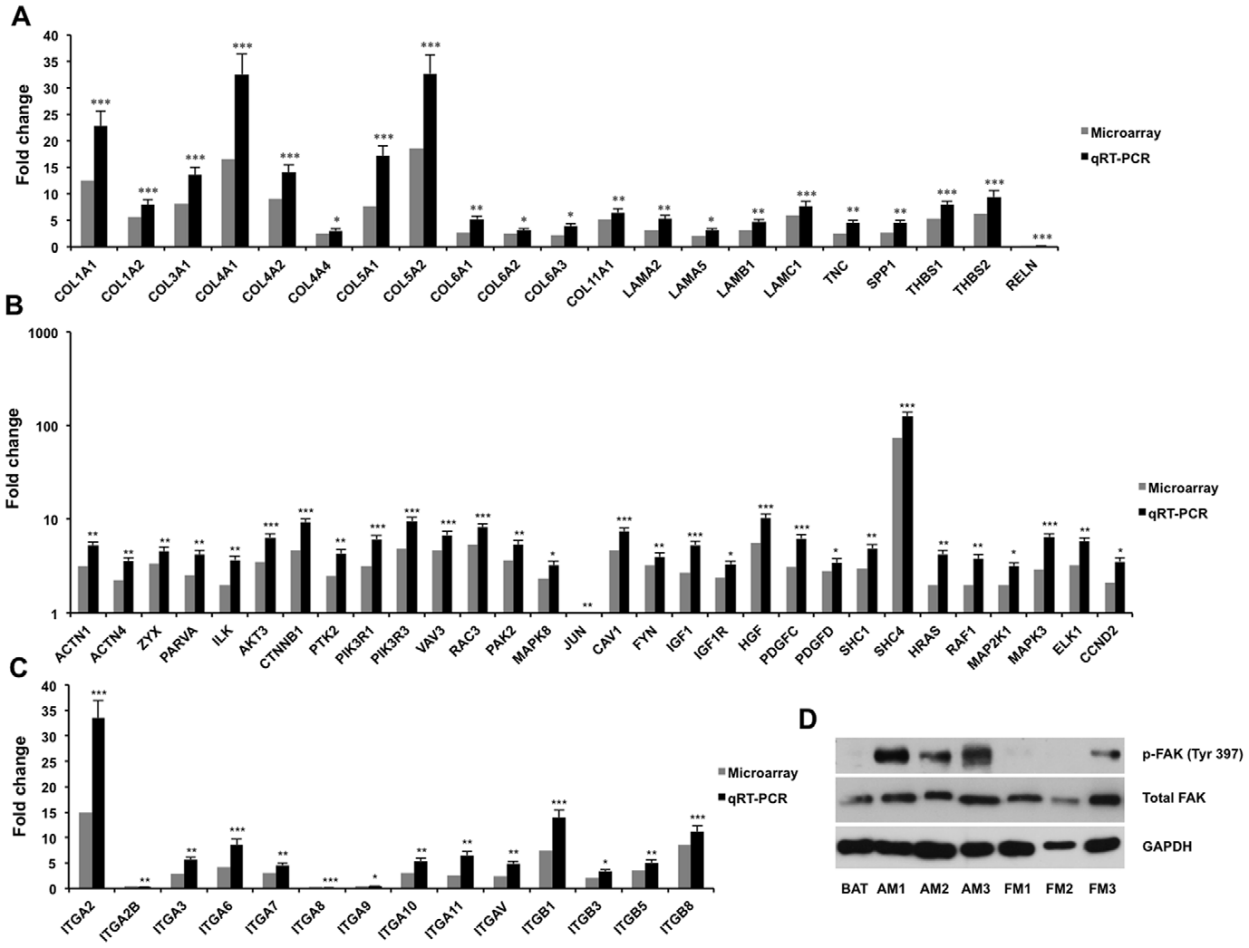
Expression of genes/proteins associated with the KEGG pathways in anaplastic meningiomas and arachnoidal tissue. qRT-PCR experiments were performed to confirm the expression of genes in 6 anaplastic meningiomas (including the 3 tested samples in microarray) and 3 arachnoidal tissues. When expression of the genes in arachnoidal tissue was set to 1, relative expression of genes of ECM family (A) and integrins (C), as well as other genes (B) in the pathway is shown in each graph by using microarray and qRT-PCR. *P<0.05, **P<0.01, ***P<0.001. Western blotting determined total FAK and phosphorylated FAK (p-FAK Tyr 397) in anaplastic, fibroblastic meningiomas and arachnoidal tissue (D). GAPDH expression was used as controls. Key words: BAT, brain arachnoidal tissue; AM, anaplastic meningioma; FM, fibroblastic meningioma.

We also performed qRT-PCRs to validate expression of these differentially expressed genes in the two pathways. We first investigated expression of genes belonging to the extracellular matrix (ECM) family between tumor and arachnoidal tissue. All of investigated ECM genes except *RELN* were found with significantly higher expression in anaplastic meningiomas than in arachnoidal tissue (Figure 3A). Notably, collagen genes including *COL1A1* (22.8-fold), *COL1A2* (7.98-fold), *COL3A1* (13.6-fold), *COL4A1* (32.57-fold), *COL4A2* (14.1-fold), *COL4A4* (3.04-fold), *COL5A1* (17.23-fold), *COL5A2* (32.73-fold), *COL6A1* (5.26-fold), *COL6A2* (3.12-fold), *COL6A3* (3.95-fold), and *COL11A1* (6.48-fold), were all up-regulated in tumor tissues by qRT-PCR (Figure 3A). Of ECM receptors, integrin family genes were detected with significantly differential expression between tumor and arachnoidal tissue. qRT-PCR analysis showed up-regulation of most of integrins in tumor tissues, including *ITGA2* (33.5-fold), *ITGA3* (5.58-fold), *ITGA6* (8.58-fold), *ITGA7* (4.57-fold), *ITGA10* (5.34-fold), *ITGA11* (6.52-fold), *ITGAV* (4.87-fold), *ITGB1* (13.95-fold), *ITGB3* (3.34-fold), *ITGB5* (5.05-fold), and *ITGB8* (11.21-fold) (Figure 3C). It was observed that multiple gene sets involved in signaling pathways downstream of integrin were expressed increasingly in tumor tissues. It is well established that integrin clustering and actin polymerization are required for FAK activation, which in turn results in cascades of protein interactions that transduce signals to many downstream pathways, including Ras/ERK, PI3K/Akt, and Crk/Dock180/Rac [13]. In our study, *FAK* (*PTK2*) gene expression was found significantly increased 4.32-fold in anaplastic meningiomas compared with arachnoidal tissue by qRT-PCR (Figure 3B), and both expression and phosphorylation of FAK protein evidently enhanced in anaplastic meningiomas when compared with either brain arachnoidal tissue or fibroblastic meningiomas (Figure 3D). Furthermore, several genes participating in these downstream pathways of integrin/FAK were also found with significantly higher expression in tumor tissues than in arachnoidal tissue, including *AKT3* (6.38-fold), *PIK3R1* (6.12-fold), *PIK3R3* (9.57-fold), *CTNNB1* (9.25-fold), *VAV3* (6.78-fold), *RAC3* (8.16-fold), *PAK2* (5.35-fold), *SHC1* (4.89-fold), *SHC4* (125-fold), *HRAS* (4.24-fold), *RAF1* (3.78-fold), *MAP2K1* (3.14-fold), *MAPK3* (6.45-fold), *ELK1* (5.78-fold) (Figure 3B). Besides ECM-integrin interaction pathway, several growth factors and their receptors were also observed up-regulated in anaplastic meningioma. It was found that expression of *IGF1*, *HGF*, *PDGFC*, and *PDGFD* were significantly increased 5.32-, 10.21-, 6.24-, and 3.24-fold in tumor tissues, respectively, when compared with arachnoidal tissue by qRT-PCR (Figure 3B). As for their receptors, *IGF1R* expression enhanced 3.28-fold in tumor tissues (Figure 3B). Taken together, the data suggested that up-regulation of the growth factor-receptor interaction played a role in tumorigenesis of anaplastic meningioma through downstream Ras/ERK or PI3K/Akt pathway.

### *EGFL6* mRNA levels in brain arachnoidal tissue and various tumors

**Figure 4:**
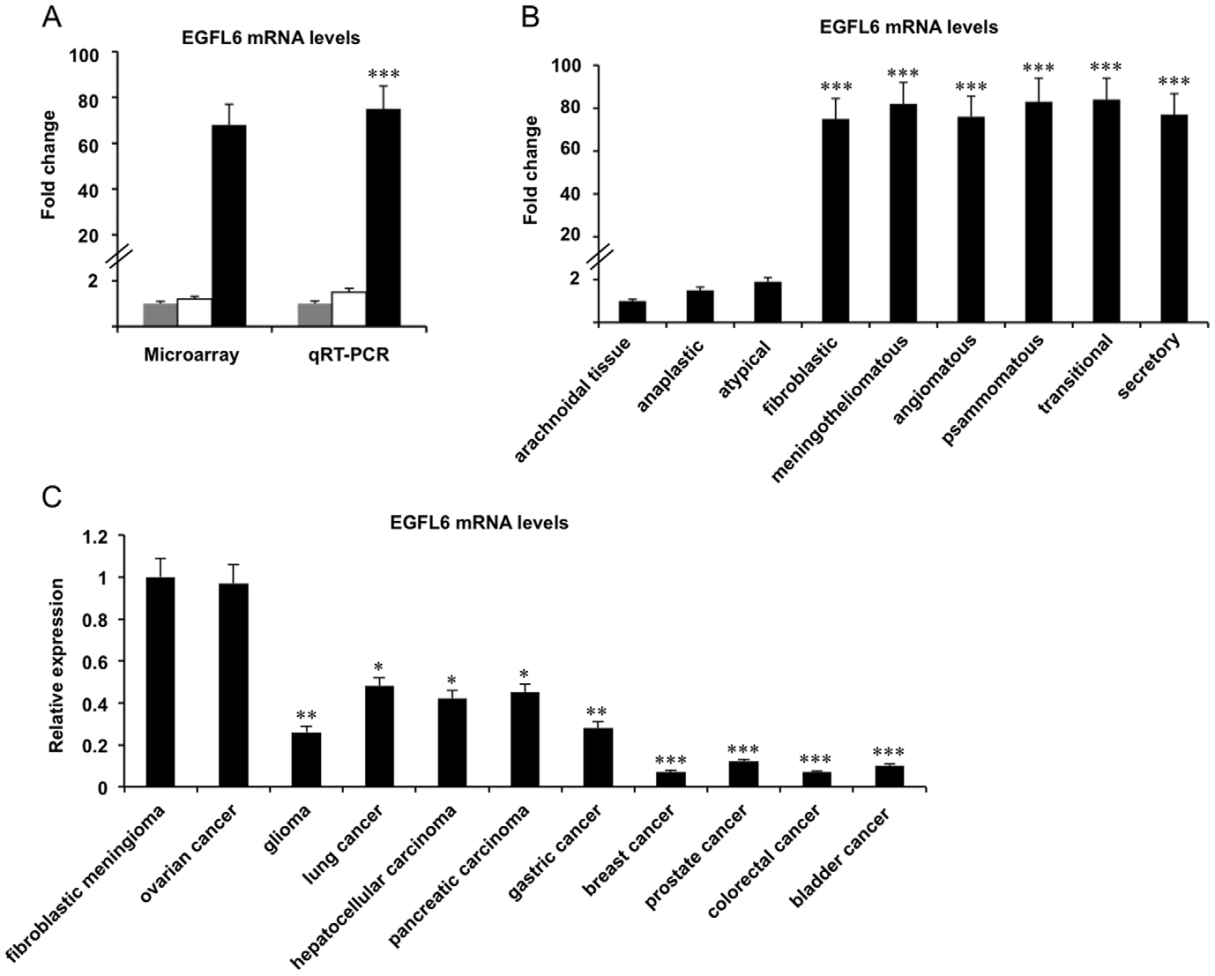
*EGFL6* gene expression in brain arachnoidal tissue and various tumors. (A) *EGFL6* gene expression in brain arachnoidal tissue (grey bars) (n = 3), tissues of anaplastic (white bars) (n = 6) and fibroblastic (black bars) (n = 10) meningiomas by microarray and qRT-PCR. *EGFL6* mRNA levels in tissues of anaplastic and fibroblastic meningiomas are shown in each graph when the *EGFL6* gene expression in brain arachnoidal tissue is set to 1. ***P<0.001. (B) *EGFL6* mRNA levels in meningiomas with different histological types are shown in each graph when the *EGFL6* gene expression in brain arachnoidal tissue is set to 1. Each tumor had 6 different samples investigated, except fibroblastic meningioma with 10. ***P<0.001. (C) *EGFL6* mRNA levels in various tumors are shown in each graph when the *EGFL6* gene expression in fibroblastic meningioma is set to 1. Each tumor had 6 different samples investigated. *P<0.05, **P<0.01, ***P<0.001.

EGFL6 was identified as one of the most up-regulated genes in fibroblastic meningioma by microarray analysis. Its expression was found increased 68- and 1.2-fold in fibroblastic and anaplastic meningiomas, respectively, when compared with brain arachnoidal tissue (Figure 4A). We next investigated whether *EGFL6* mRNA levels were up-regulated in meningiomas with other histological subtypes. qRT-PCR analysis indicated that besides fibroblastic meningioma, other subtype benign meningioma also had high levels of *EGFL6* mRNA. When compared with brain arachnoidal tissue, 82- (*P*<0.001), 75- (*P*<0.001), 83- (*P*<0.001), 84- (*P*<0.001), and 77-fold (*P*<0.001) increases of *EGFL6* mRNA levels were observed in meningotheliomatous, angiomatous, psammomatous, transitional, and secretory meningiomas, respectively (Figure 4B). However, *EGFL6* mRNA was detected with low levels in atypical meningiomas (1.9-fold, vs brain arachnoidal tissue) (Figure 4B).

We expanded the survey of *EGFL6* gene expression profile in other tumor tissues. Interestingly, *EGFL6* mRNA was also found with a high level in ovarian cancer, comparable to that in benign meningioma (Figure 4C). When compared with fibroblastic meningioma, *EGFL6* mRNA levels were significantly decreased in all other investigated tumors including glioma (3.84-fold, *P*<0.01), lung cancer (2.08-fold, *P*<0.05), hepatocellular carcinoma (2.38-fold, *P*<0.05), pancreatic carcinoma (2.22-fold, *P*<0.05), and gastric cancer (3.57-fold, *P*<0.01), breast cancer (14.29-fold, *P*<0.001), prostate cancer (8.33-fold, *P*<0.001), colorectal cancer (14.23-fold, *P*<0.001), and bladder cancer (10-fold, *P*<0.001) (Figure 4C).

### Serum EGFL6 levels in healthy controls and patients with various tumors

**Figure 5:**
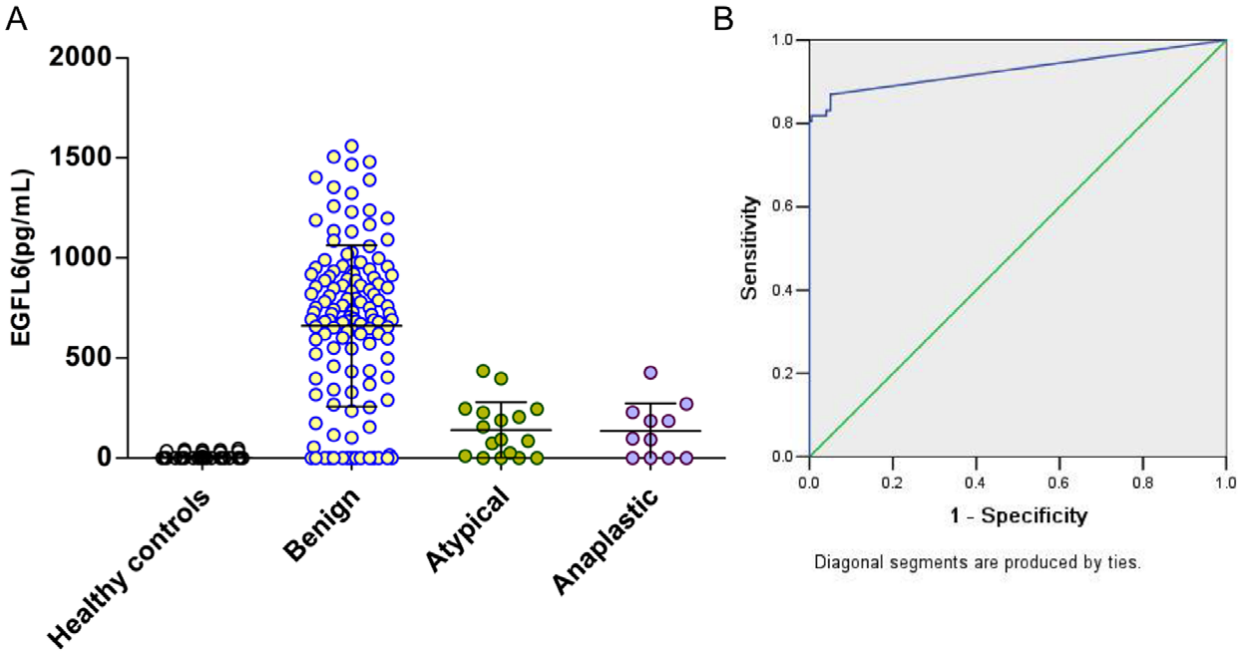
Serum EGFL6 levels in patients with meningiomas. (A) Scatter plot presentation of serum EGFL6 concentrations in patients with meningioma and healthy controls. Black horizontal lines are means. (B) ROC curve for serum EGFL6 levels in meningioma.

Serum EGFL6 concentrations were evaluated by using ELISA. The serum EGFL6 concentrations were listed in Table 1. Firstly, we investigated serum EGFL6 levels in patients with meningiomas. The mean serum EGFL6 concentration was 672, 139, and 135 pg/ml in patients with meningiomas classified as WHO grade I (benign), grade II (atypical), and grade III (anaplastic) respectively, indicating a significant increase of serum EGFL6 level in benign meningioma patients when compared with atypical (*P*<0.001) or anaplastic meningioma patients (*P*<0.001) (Table 1). In order to show the distribution of serum EGFL6 in patients with meningioma more clearly, a scatter plot graph was generated (Figure 5A). We performed ROC analysis to evaluate the sensitivity and specificity of serum EGFL6 levels for meningioma. The sensitivity of serum EGFL6 levels in patients with meningioma relative to healthy controls was 81%, and the specificity was 100% at a cutoff value of 64.38 pg/ml (Figure 5B). The area under the curve for serum EGFL6 was 0.912±0.018 (*P*<0.001) (Figure 5B). There were no significant differences of serum EGFL6 concentrations among patients with benign meningiomas including fibroblastic, meningotheliomatous, angiomatous, psammomatous, transitional, and secretory meningiomas (Figure S3A). The mean serum EGFL6 concentration in the female patients with benign meningioma was slightly higher than that in male patients, but the difference was not significant (Figure S3B). We also investigated whether serum EGFL6 concentrations were correlated with tumor sizes. It was found that serum EGFL6 concentrations in patients with benign meningiomas were not significantly correlated with tumor sizes quantified by magnetic resonance imaging (MRI) (r = −0.1964, *P* = 0.2896). Neither were the correlations observed in patients with atypical or anaplastic meningiomas.

**Table 1:** Serum EGFL6 levels in healthy controls and patients with various tumors.

	Positivity (%)	Range (pg/ml)	Mean concentration (pg/ml)	*P* value (*vs.* benign meningioma)
Healthy controls	5.5	0–48	2	<0.001
**Meningiomas**				
Benign (n = 132)	84	0–1560	672	
Atypical (n = 17)	64.7	0–436	139	<0.001
Anaplastic (n = 11)	63.6	0–428	135	<0.001
**Other tumors**				
Glioma (n = 52)	65.4	0–465	161	<0.001
Pituitary adenoma (n = 56)	67.9	0–527	215	<0.01
Acoustic neuroma (n = 16)	62.5	0–318	121	<0.001
Craniopharyngioma (n = 24)	62.5	0–367	151	<0.001
Cavernous hemangioma (n = 23)	61	0–383	148	<0.001
Schwannoma (n = 30)	56.7	0–371	137	<0.001
Ovarian cancer (n = 50)	80	0–1364	616	0.862
Gastric cancer (n = 48)	56.25	0–432	137	<0.001
Breast cancer (n = 42)	42.86	0–189	61	<0.001
Pancreatic carcinoma (n = 33)	66.7	0–705	232	<0.01
Prostate cancer (n = 40)	55	0–258	98	<0.001
Hepatocellular carcinoma (n = 45)	66.7	0–452	202	<0.001
Lung cancer (n = 51)	72.5	0–486	216	<0.01
Colorectal cancer (n = 36)	52.8	0–164	92	<0.001
Bladder cancer (n = 43)	51.16	0–278	97	<0.001

We next investigated serum EGFL6 levels in other tumors. Importantly, patients with ovarian cancer also had high serum EGFL6 levels (mean concentration: 616 pg/ml) (Table 1). As for patients with other tumors such as gastric cancer, breast cancer, pancreatic carcinoma, prostate cancer, hepatocellular carcinoma, lung cancer, colorectal cancer, bladder cancer, gliomas, pituitary adenomas, acoustic neuromas, craniopharyngiomas, cavernous hemangiomas, and schwannomas, their serum EGFL6 concentrations were all significantly lower than those of patients with benign meningioma (Table 1).

## Discussion

In the present study, we established differential gene expression profiles between brain arachnoidal tissue and fibroblastic meningioma, and also between brain arachnoidal tissue and anaplastic meningioma. Through analysis of the microarray results, several signaling pathways associated with tumorigenesis of meningioma were generated and EGFL6 was found overexpressed in benign meningioma tissues and serum.

We proposed PI3K/Akt, cell cycle, and TGFβ signaling pathways as candidate pathways involved in the pathogenesis of fibroblastic meningioma. We found that several growth factors including *EGFL6*, *IGF1*, *FGF2*, *FGF7*, *FGF9*, and *FGF11* were overexpressed in fibroblastic meningioma, especially *EGFL6* gene with extremely high expression in benign meningioma tissues. Growth factor stimulation is required for tumor cell proliferation. Many cancer cells acquire the ability to synthesize growth factors to which they are responsive, creating a positive feedback signaling loop often termed autocrine stimulation. The production of PDGF (platelet-derived growth factor) and TGFα (tumor growth factor α) by glioblastomas and sarcomas, respectively, are two illustrative examples [14]. PI3K/Akt signaling cascade is a well-known downstream pathway of growth factor-receptor interaction. It is now known that PI3K/Akt signaling pathway can be activated by a diverse array of physiologic stimuli including EGF, IGF, and FGF [15]. We observed that both *PI3K* (*PIK3R1*, *PIK3R3*) and *Akt* (*AKT3*) genes were up-regulated in fibroblastic meningioma, and total Akt and phosphorylation Akt were also overexpressed in tumor tissues. With respect to downstream gene sets of PI3K/Akt pathway, proliferation-promoted genes such as *CTNNB1*, *CREB1*, *CREB5*, and *TCF7L2* were up-regulated in fibroblastic meningioma. However, apoptosis-induced genes such as *CASP9*, *CDKN1A*, *AXIN2*, *APC2*, and *EP300* were down-regulated. Our results were consistent with another study on predicted pathways in meningioma tumorigenesis, in which activation of PI3K/Akt pathway was observed in meningiomas, but not in normal brain tissues [16]. It has been established that the PI3K/Akt pathway, which transmits anti-apoptotic survival signals, is involved in mitigating apoptosis in a substantial fraction of human tumors [15], [17]. Taken together, it suggested that up-regulation of PI3K/Akt signaling pathway played a role in the tumorigenesis of benign meningioma via promoting cell proliferation and inhibiting apoptosis. Activation of Ras/ERK pathway has been regard as one of the most important events in the growth factor signaling. We noticed that several components of Ras/ERK pathway were overexpressed in fibroblastic meningioma, including *SHC4*, *SOS2*, *KRAS*, and *CCDC6*. However, c-Jun and c-Fos, two critical downstream effectors of the pathway, were detected with reduced mRNA levels in tumor tissues. It is now clear that there exits a cross-talking connection between Ras/ERK and PI3K/Akt pathway via the direct interaction of Ras and PI3K [14], [15]. Considering inconsistent expression of *SOS2/KRAS* and *JUN/FOS* in fibroblastic meningioma, we speculated that activated Ras promoted tumor growth through the PI3K/Akt pathway, but not via ERK-c-Jun/c-Fos pathway. Besides PI3K/Akt pathway, TGFβ and cell cycle pathways were also proposed to be associated with fibroblastic meningioma. Since several key genes in the pathway were overexpressed in tumor tissues, including *TGFB2*, *TGFBR1*, *SMAD2*, and *SMAD4*, TGFβ signaling pathway may be involved in the pathogenesis of fibroblastic meningioma. Cell cycle deregulation is a common feature of human cancer. The mammalian cell cycle is controlled by a subfamily of cyclin-dependent kinases (CDKs), the activity of which is modulated by several activators and inhibitors. Deregulation of CDKs and their inhibitors has been implicated in a wide variety of tumors [18]–[20]. In our study, fibroblastic meningioma was detected with increasing expression of *CCND1*, *CDK6*, and *E2F3*, and decreasing expression of CDKs inhibitors including *CDKN1A*, *CDKN2A*, and *CDKN2B*, suggesting a role of cell cycle deregulation in the tumorigenesis of fibroblastic meningioma.

Anaplastic meningioma is characteristic by invasive growth, high recurrence rate, and short survival time, which differs extensively from benign meningioma [1]. Overexpression of ECM, integrin family genes and their downstream cell adhesion molecules suggested that activation of integrin-mediated cell adhesion signaling pathway was involved in the pathogenesis of anaplastic meningioma. Noticeably, our result was far from that of the study by Keller et al, in which signaling molecules in pathways of cell adhesion and ECM-receptor interaction were found with decreasing expression in meningioma [21]. We considered that the contradictory result might be caused by different control tissues used in the two studies (arachnoidal tissue in our study, and dura tissue in Keller's study). Furthermore, the histological type of meningioma compared to dura tissue is unspecific in the study by Keller et al [21]. ECM plays a significant role in regulating many cellular behaviors, such as cell shape, adhesion, migration, proliferation, polarity, differentiation and apoptosis. In numerous tumors, increased synthesis of certain ECM components contributes to tumor growth, invasion, and metastasis. We observed that many ECM components, especially a cluster of collagens, had overexpression at mRNA levels in anaplastic meningioma, similar with another study on meningiomas in which the tumors show higher expression of some types of collagen [16]. The effects of ECM on cells are mainly mediated by integrins, a large family of cell-surface receptors. Changes in integrin expression are evident in invasive and metastatic cells [14]. We found that up-regulation of integrins predominated in anaplastic meningioma. It is increasingly clear that neoplastic cells enhance the expression of integrins that favor their proliferation, survival, and migration [22]. Concordant with the up-regulation of ECM and integrins, cascades of genes associated with the downstream pathways of integrin were also found with increasing expression, indicating up-regulation of integrin-mediated intracellular signaling pathways in anaplastic meningioma. Most of integrins activate FAK and thereby the downstream signaling pathways including Ras/ERK, PI3K/Akt, and Crk/Dock180/Rac pathways. Studies on FAK-null fibroblasts and keratinocyte cells have shown that FAK is crucial for cell migration and tumor growth [23], [24]. It is well addressed that many invasive human cancers have elevated levels of FAK [25]. Notably, we also detected overexpression of FAK gene and enhanced phosphorylated and total FAK protein in anaplastic meningioma. Numerous observations show that the integrin-activated pathways are sufficient to induce cell migration, to elevate cytoskeletal tension, and to confer some protection from apoptosis on cells [22], [26]. Based on these observations, up-regulation of the integrin-mediated signaling pathways were implicated in the cell proliferation and migration, and tumor metastasis of anaplastic meningioma.

Another important result of our study is to find EGFL6 overexpression in benign meningioma tissues and serum. *EGFL6*, also known as *MAEG*, is highly expressed in fetal tissues and meningioma [27]–[30]. We observed that *EGFL6* mRNA levels were significantly increased in benign meningioma when compared with either brain arachnoidal tissue or atypical or anaplastic meningiomas. Previous evidence demonstrated that *EGFL6* transcript was detected in two meningiomas, and not observed in a glioma and a malignant lymphoma [27]. We also found that *EGFL6* gene was expressed at a low level in all other tumors except ovarian cancer. *EGFL6* mRNA levels were very high in ovarian cancer, which were comparable with benign meningioma. The result was concordant with the data presented by Buckanovich et al [31]. In that study, *EGFL6* showed limited or no expression in normal tissues by qRT-PCR and analysis of a publicly available gene expression profile data set, but was expressed at a high level in ovarian cancer. In another report, *EGFL6* gene expression was observed significantly increased in tumor-associated endothelial cells in comparison with their normal counterparts [32]. Besides benign meningioma and ovarian cancer, *MAEG* (*EGFL6*) gene was also found expressed in several fetal tissues including lung, kidney, dermatome and dermatome derivatives [28], [29], [33]. Taken together, *EGFL6* gene expression profile has been established by several lines of studies. Our study enriched the *EGFL6* gene expression profile by determining *EGFL6* mRNA levels in meningioma with different WHO grades and subtypes and extending the survey of *EGFL6* mRNA levels in tumor tissues.

A putative signal peptide is predicted at the N terminal of EGFL6 protein, suggesting that the protein is secreted [27], [34]. It has been reported that EGFL6 can be detectable in medium culturing *EGFL6*-overexpressed COS-7 cells by using western blotting [30]. Although Oberauer et al [35] had established direct ELISA to detect EGFL6 protein in culture medium, quantitative analysis of serum EGFL6 has not been reported until now. In this study, we measured human serum EGFL6 by ELISA. We found that EGFL6 protein was hardly detected in serum of healthy controls, and also was low in most of tumors, which agreed with its mRNA levels in normal adult tissues and tumor tissues. Patients with benign meningiomas and ovarian cancers had significantly higher serum EGFL6 levels than those with other intra- and extra-cranial tumors. Among different WHO grades of meningiomas, it was significant that serum EGFL6 was detected with higher levels in patients with benign meningiomas than those with atypical and anaplastic tumors. Although high levels of EGFL6 in benign meningioma, due to small sizes of atypical and anaplastic tumors in our study, it is not easy to use EGFL6 to help discriminate benignancy or malignancy of meningioma before surgery or at early time points. ROC analysis indicated high sensitivity (81%) and high specificity (100%) of serum EGFL6 for patients with meningioma. However, we have realized that this study had limitations in the population of healthy people and patients with other tumors. We are very careful in describing EGFL6 as a biomarker for meningioma. So future studies on EGFL6 serology will be focused on enlarging the population and types of tumors or illnesses as many as possible, by which a more precise distribution of EGFL6 in human serum is determined.

The role of EGFL6 in the pathogenesis of benign meningioma remained unknown. Upon binding to their receptors, EGF-like repeat family members are often involved in the regulation of cell cycle, proliferation, apoptosis, differentiation, adhesion and migration [36]. Recent evidence indicates that EGFL6 induces cell migration and angiogenesis of endothelial cells via activation of ERK pathway [30]. Another study on mouse hair follicle morphogenesis showed that EGFL6 may play a role as a mediator regulating epithelial–mesenchymal interaction through binding to RGD-binding integrins including α8β1 during hair follicle development [37]. It has been established that EGF family factors can activate PI3K/Akt signaling pathway [15]. Considering overexpression of EGFL6 and activation of PI3K/Akt pathway in fibroblastic meningioma, we speculated that EGFL6 played a role in the pathogenesis of benign meningioma via activating PI3K/Akt pathway.

In conclusion, we have established differential gene expression profiles between brain arachnoidal tissue and fibroblastic and anaplastic meningiomas. KEGG pathway analysis suggested that activation of the PI3K/Akt and the integrin-mediated cell adhesion signaling pathways played important roles in the pathogenesis of benign and anaplastic meningiomas, respectively. We presented evidence that patients with benign meningioma had high levels of tissue EGFL6 mRNA and serum EGFL6 concentration.

## Supporting Information

Figure S1 **Schematic model of KEGG pathways in fibroblastic meningioma.** Up-regulated genes are shown in red, and down-regulated genes in blue. Genes that were not differentially expressed are shown in black.
(TIF)

Figure S2 **Schematic model of KEGG pathways in anaplastic meningioma.** Up-regulated genes are shown in red, and down-regulated genes in blue. Genes that were not differentially expressed are shown in black.
(TIF)

Figure S3 **The mean serum EGFL6 concentration (MSEC) in patients with benign meningiomas.** (A) MSEC in patients with different subtypes including fibroblastic (n = 61), meningotheliomatous (n = 25), angiomatous (n = 11), psammomatous (n = 10), transitional (n = 10), and secretory meningiomas (n = 15). (B) MSEC in female (n = 83) and male (n = 49) patients with benign meningiomas.
(TIF)

Table S1 **Significant KEGG pathways detected based on the differentially expressed genes between fibroblastic meningioma and brain arachnoidal tissue.**
(DOC)

Table S2 **Significant KEGG pathways detected based on the differentially expressed genes between anaplastic meningioma and brain arachnoidal tissue.**
(DOC)
