# Supplementary figures and images for: Analysis of Gene Expression Profiling in Meningioma: Deregulated Signaling Pathways Associated with Meningioma and EGFL6 Overexpression in Benign Meningioma Tissue and Serum
Source: PLoS One. 2012 Dec 28;7(12):e52707. doi: 10.1371/journal.pone.0052707 (PMC3532066; doi:10.1371/journal.pone.0052707)

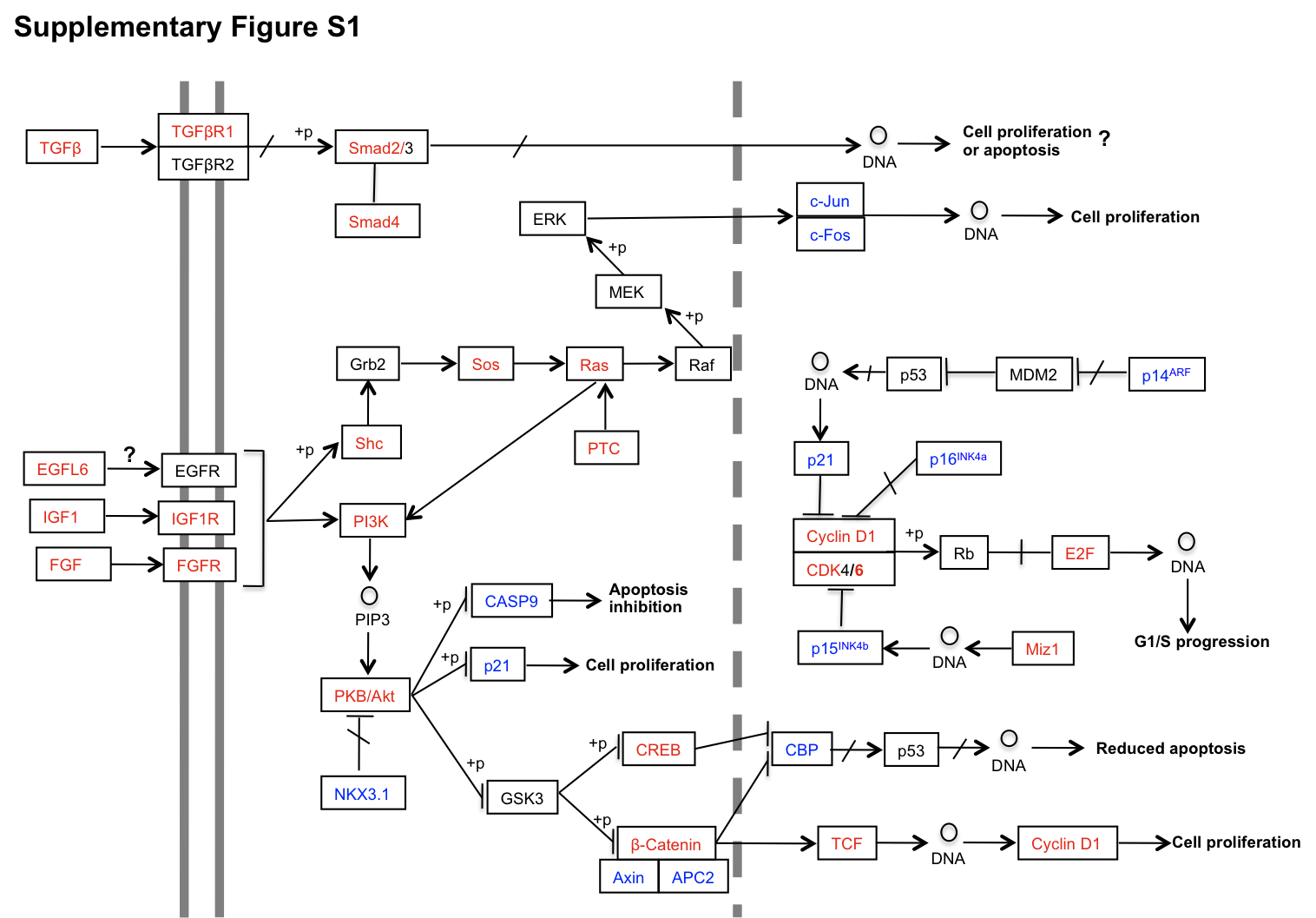

Supplement: Figure S1 — Schematic model of KEGG pathways in fibroblastic meningioma. Up-regulated genes are shown in red, and down-regulated genes in blue. Genes that were not differentially expressed are shown in black. (TIF) [file pone.0052707.s001.tif]

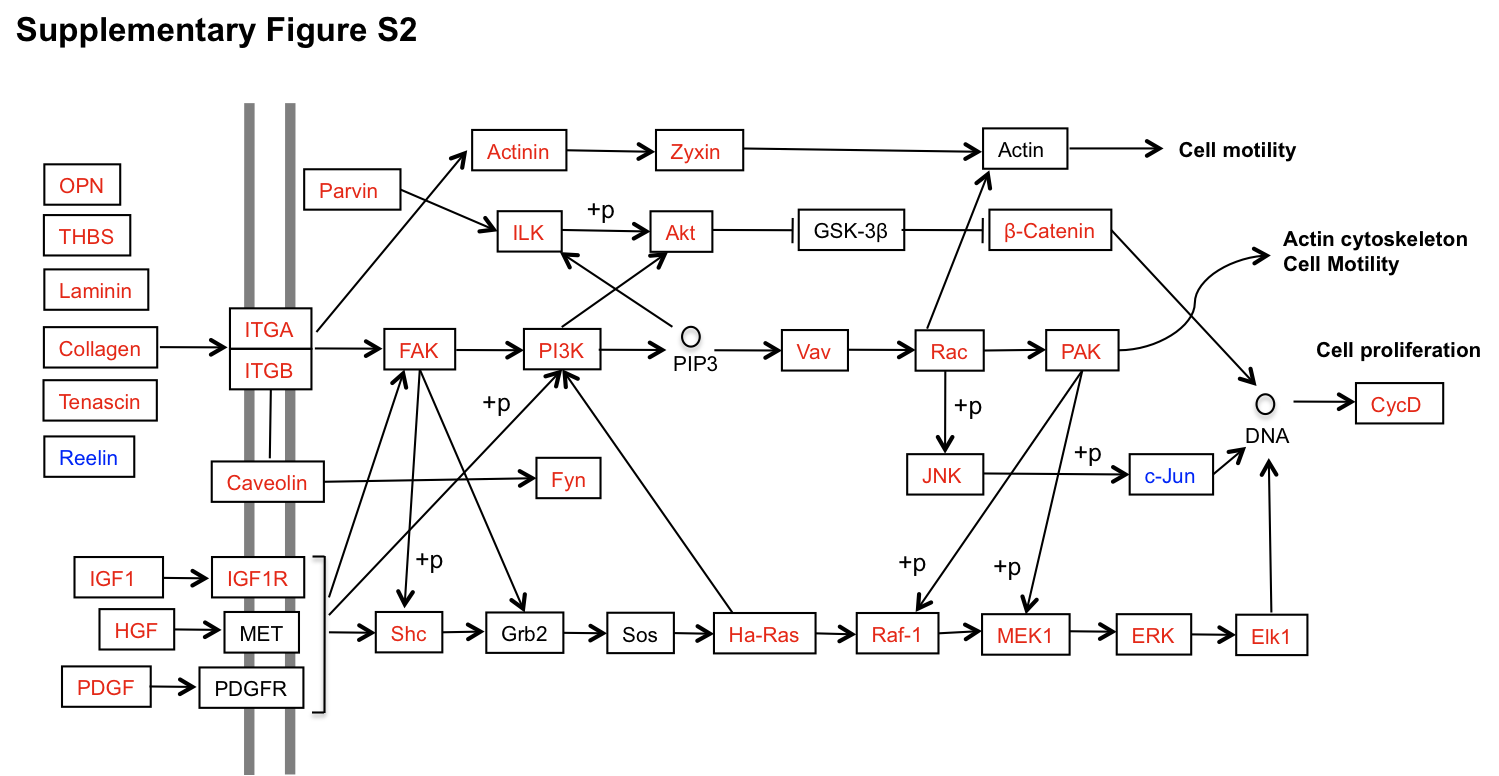

Supplement: Figure S2 — Schematic model of KEGG pathways in anaplastic meningioma. Up-regulated genes are shown in red, and down-regulated genes in blue. Genes that were not differentially expressed are shown in black. (TIF) [file pone.0052707.s002.tif]

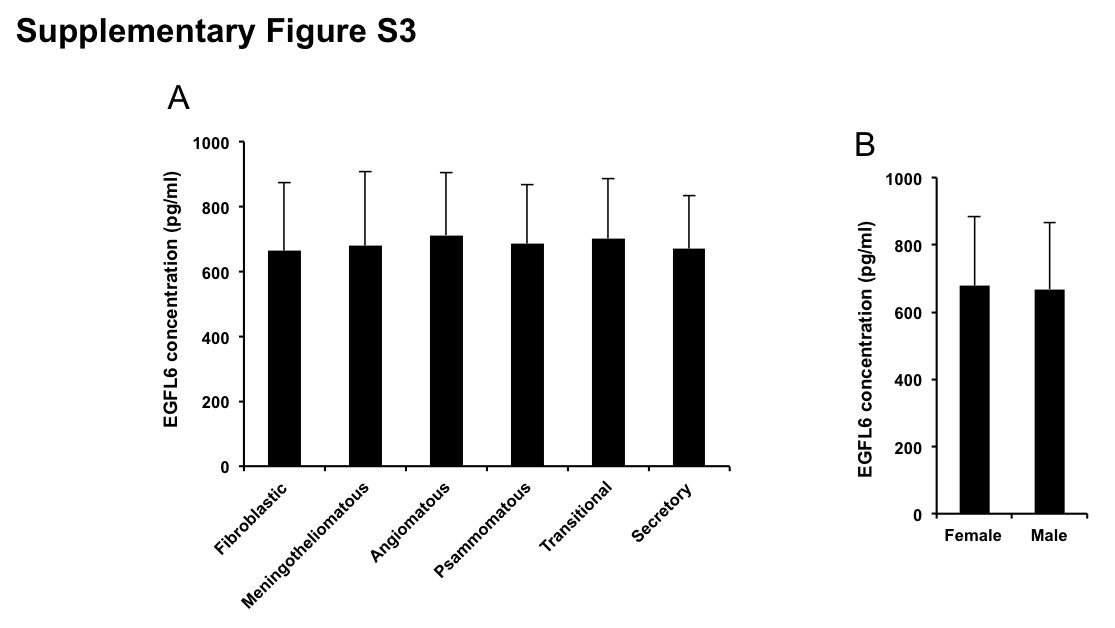

Supplement: Figure S3 — The mean serum EGFL6 concentration (MSEC) in patients with benign meningiomas. (A) MSEC in patients with different subtypes including fibroblastic (n = 61), meningotheliomatous (n = 25), angiomatous (n = 11), psammomatous (n = 10), transitional (n = 10), and secretory meningiomas (n = 15). (B) MSEC in female (n = 83) and male (n = 49) patients with benign meningiomas. (TIF) [file pone.0052707.s003.tif]
