# Supplementary material for: Analysis of Gene Expression Profiling in Meningioma: Deregulated Signaling Pathways Associated with Meningioma and EGFL6 Overexpression in Benign Meningioma Tissue and Serum
Source: PLoS One. 2012 Dec 28;7(12):e52707. doi: 10.1371/journal.pone.0052707 (PMC3532066; doi:10.1371/journal.pone.0052707)
Supplement: Table S1 — Significant KEGG pathways detected based on the differentially expressed genes between fibroblastic meningioma and brain arachnoidal tissue. (DOC) [file pone.0052707.s004.doc]

Table S1. Significant KEGG pathways detected based on the differentially expressed genes between fibroblastic meningioma and brain arachnoidal tissue

| **KEGG ID** | **Term** | **Gene Count** | **Percent** | **P Value** |
| --- | --- | --- | --- | --- |
| hsa05200 | Pathways in cancer | 91 | 2.6963 | 3.06E-05 |
| hsa04120 | Ubiquitin mediated proteolysis | 44 | 1.3037 | 1.79E-04 |
| hsa04510 | Focal adhesion | 57 | 1.6889 | 6.66E-04 |
| hsa04310 | Wnt signaling pathway | 45 | 1.3333 | 9.12E-04 |
| hsa04960 | Aldosterone-regulated sodium reabsorption | 17 | 0.5037 | 0.001726 |
| hsa04010 | MAPK signaling pathway | 69 | 2.0444 | 0.002591 |
| hsa04360 | Axon guidance | 38 | 1.1259 | 0.003068 |
| hsa05215 | Prostate cancer | 28 | 0.8296 | 0.004675 |
| hsa04610 | Complement and coagulation cascades | 23 | 0.6815 | 0.005332 |
| hsa05218 | Melanoma | 23 | 0.6815 | 0.007748 |
| hsa05210 | Colorectal cancer | 26 | 0.7704 | 0.008295 |
| hsa04115 | p53 signaling pathway | 22 | 0.6519 | 0.009546 |
| hsa05410 | Hypertrophic cardiomyopathy (HCM) | 26 | 0.7704 | 0.009739 |
