# Supplementary material for: Analysis of Gene Expression Profiling in Meningioma: Deregulated Signaling Pathways Associated with Meningioma and EGFL6 Overexpression in Benign Meningioma Tissue and Serum
Source: PLoS One. 2012 Dec 28;7(12):e52707. doi: 10.1371/journal.pone.0052707 (PMC3532066; doi:10.1371/journal.pone.0052707)
Supplement: Table S2 — Significant KEGG pathways detected based on the differentially expressed genes between anaplastic meningioma and brain arachnoidal tissue. (DOC) [file pone.0052707.s005.doc]

Table S2. Significant KEGG pathways detected based on the differentially expressed genes between anaplastic meningioma and brain arachnoidal tissue

| **KEGG ID** | **Term** | **Gene Count** | **Percentage** | **P Value** |
| --- | --- | --- | --- | --- |
| hsa04510 | Focal adhesion | 66 | 2.1229 | 2.35E-07 |
| hsa04512 | ECM-receptor interaction | 35 | 1.1258 | 7.10E-07 |
| hsa04115 | p53 signaling pathway | 26 | 0.8363 | 1.39E-04 |
| hsa05412 | Arrhythmogenic right ventricular cardiomyopathy (ARVC) | 28 | 0.9006 | 1.49E-04 |
| hsa04120 | Ubiquitin mediated proteolysis | 41 | 1.3188 | 5.86E-04 |
| hsa05410 | Hypertrophic cardiomyopathy (HCM) | 28 | 0.9006 | 0.001133 |
| hsa05200 | Pathways in cancer | 80 | 2.5732 | 0.001706 |
| hsa04520 | Adherens junction | 25 | 0.8041 | 0.002779 |
| hsa04110 | Cell cycle | 36 | 1.1579 | 0.002786 |
| hsa04012 | ErbB signaling pathway | 27 | 0.8684 | 0.003622 |
| hsa04610 | Complement and coagulation cascades | 22 | 0.7076 | 0.006758 |
| hsa05222 | Small cell lung cancer | 25 | 0.8041 | 0.009333 |
